# Seasonal variation of total and bioavailable 25-hydroxyvitamin D [25(OH)D] in the healthy adult Slovenian population

**DOI:** 10.3389/abp.2024.13108

**Published:** 2024-09-11

**Authors:** Joško Osredkar, Vid Vičič, Maša Hribar, Evgen Benedik, Darko Siuka, Aleš Jerin, Urška Čegovnik Primožič, Teja Fabjan, Kristina Kumer, Igor Pravst, Katja Žmitek

**Affiliations:** ^1^ Clinical Institute of Clinical Chemistry and Biochemistry, University Medical Centre Ljubljana, Ljubljana, Slovenia; ^2^ Faculty of Pharmacy, University of Ljubljana, Ljubljana, Slovenia; ^3^ Faculty of Health Sciences, Biomedicine in Healthcare, University of Ljubljana, Ljubljana, Slovenia; ^4^ Nutrition Institute, Ljubljana, Slovenia; ^5^ Biotechnical Faculty, Department of Food Science and Technology, Group for Nutrition, Ljubljana, Slovenia; ^6^ Division of Paediatrics, University Medical Centre Ljubljana, Ljubljana, Slovenia; ^7^ Division of Internal Medicine, Department of Gastroenterology, University Medical Centre Ljubljana, Ljubljana, Slovenia; ^8^ Faculty of Applied Sciences (VIST), Ljubljana, Slovenia

**Keywords:** vitamin 25(OH)D, free fraction of vitamin D, bioavailable fraction of vitamin D, seasonal variation, calculated fractions

## Abstract

**Objective:** The aim of our study was to compare the total 25(OH)D fraction, the bioavailable vitamin fraction, and the free vitamin D fraction in spring and fall in a group of healthy individuals.

**Methods:** In our study, we collected blood samples from healthy participants at the end of both summer and winter, and measured serum levels of albumin, DBP, and 25(OH)D. Utilizing these data, we calculated the percentage of free and bioavailable vitamin D. Our cohort comprised 87 participants, with a male-to-female ratio of 14:73, aged 35.95 ± 12.55 years, ranging from 19 to 70 years. We employed the chemiluminescence method to determine the vitamin 25(OH)D levels, the ELISA method was utilized to determine DBP levels, the albumin BCP Assay was performed using the ADVIA biochemical analyzer (Siemens) and an online calculator was used to determine the free and bioavailable 25(OH)D levels.

**Results:** Our findings indicate significantly lower 25(OH)D levels in winter (44.13 ± 17.82 nmol/L) compared to summer (74.97 ± 22.75 nmol/L; *p* < 0.001). For vitamin D binding protein there was no significant difference from summer (236.2 ± 164.39 mg/L) to winter (239.86 ± 141.9 mg/L; *p* = 0.77), albumin levels were significantly higher in summer (49.37 ± 4.15 g/L vs. 47.97 ± 3.91 g/L, *p* = 0.01), but the magnitude of the change may not be large enough to be solely responsible for the stability of vitamin D levels throughout the year. In the winter season a significantly lower calculated bioavailable 25(OH)D vitamin (7.45 ± 5.66 nmol/L against 13.11 ± 8.27 nmol/L; *p* < 0.001) was observed, and the free fraction also showed a significant decrease (17.3 ± 12.9 pmol/L versus 29.7 ± 19.1 pmol/L; *p* < 0.0001). We observed a moderately positive correlation between 25(OH)D and bioavailable percentage in winter (r = 0.680; *p* < 0.001), in contrast with a lower positive association in summer (r = 0.343; *p* < 0.001).

**Conclusion:** Our data suggest a positive correlation between total and bioavailable 25(OH)D levels. In addition to the statistically significant variation in 25(OH)D between the two observation periods, there was an additional variation in the free vitamin D percentage. The summertime synthesis of vitamin D in the skin could contribute directly to the free fraction of vitamin D. Standardizing the measurement of free 25(OH)D and clinical studies is necessary to establish reference values before these methods can be implemented in clinical practice.

## Introduction

### Physiological functions

Vitamin D is a pro-hormone that plays a crucial role in phosphorus-calcium metabolism and is therefore essential for the maintenance of healthy bones and teeth (Holick, 2008a).

Vitamin D is also involved in several other important physiological functions, including: 1) maintaining immune function by stimulating white blood cell production, 2) regulating cell growth and differentiation, which is important for maintaining healthy tissues and organs, 3) reducing the risk of depression and improving cognitive function, and 4) regulating gene expression through vitamin D’s involvement in the regulation of several genes important for maintaining health and preventing disease (Holick, 2008a; Haussler et al., 2008; Wacker and Holick, 2013; Bouillon and Carmeliet, 2018; Carlberg and Haq, 2018). In recent years, the status of vitamin D has received much attention in the context of COVID-19, which has also contributed to a significant increase in vitamin D supplementation compared to pre-pandemic levels (Žmitek et al., 2021; Vičič and Pandel Mikuš, 2023).

### Vitamin D insufficiency and deficiency

Vitamin D deficiency is associated with bone diseases such as rickets and osteoporosis. Low vitamin D levels are also associated with an increased risk of other chronic diseases such as cardiovascular disease, cancer and autoimmune diseases (Calvo et al., 2004; Holick, 2008b; Heaney et al., 2017).

Based on the data that are now available and clinical considerations, various associations and organizations employ slightly different criteria for vitamin D insufficiency, deficiency, and sufficiency.

The Endocrine Society recommends that vitamin D status be assessed in patients who are at risk for vitamin D deficiency by measuring the circulating serum 25(OH)D level using an accurate test. A 25(OH)D level of less than 20 ng/mL (50 nmol/L) is considered to be vitamin D insufficiency, whereas a 25(OH)D of 21–29 ng/mL (52.5–72.5 nmol/L) is considered to be vitamin D sufficiency (Holick et al., 2011).

A multidisciplinary Polish panel developed concerns regarding the guidelines for treating and preventing vitamin D insufficiency in both the general population and in high-risk patient groups. The ranges of total blood 25-hydroxyvitamin D concentration indicating suboptimal status (>20 ng/mL, <50 nmol/L), ideal concentration (>30–50 ng/mL, <75 nmol/L), and vitamin D insufficiency (<20 ng/mL, <50 nmol/L) were confirmed on the basis of network discussions (Płudowski et al., 2023).

### Vitamin D metabolism and fractions

**FIGURE 1:**
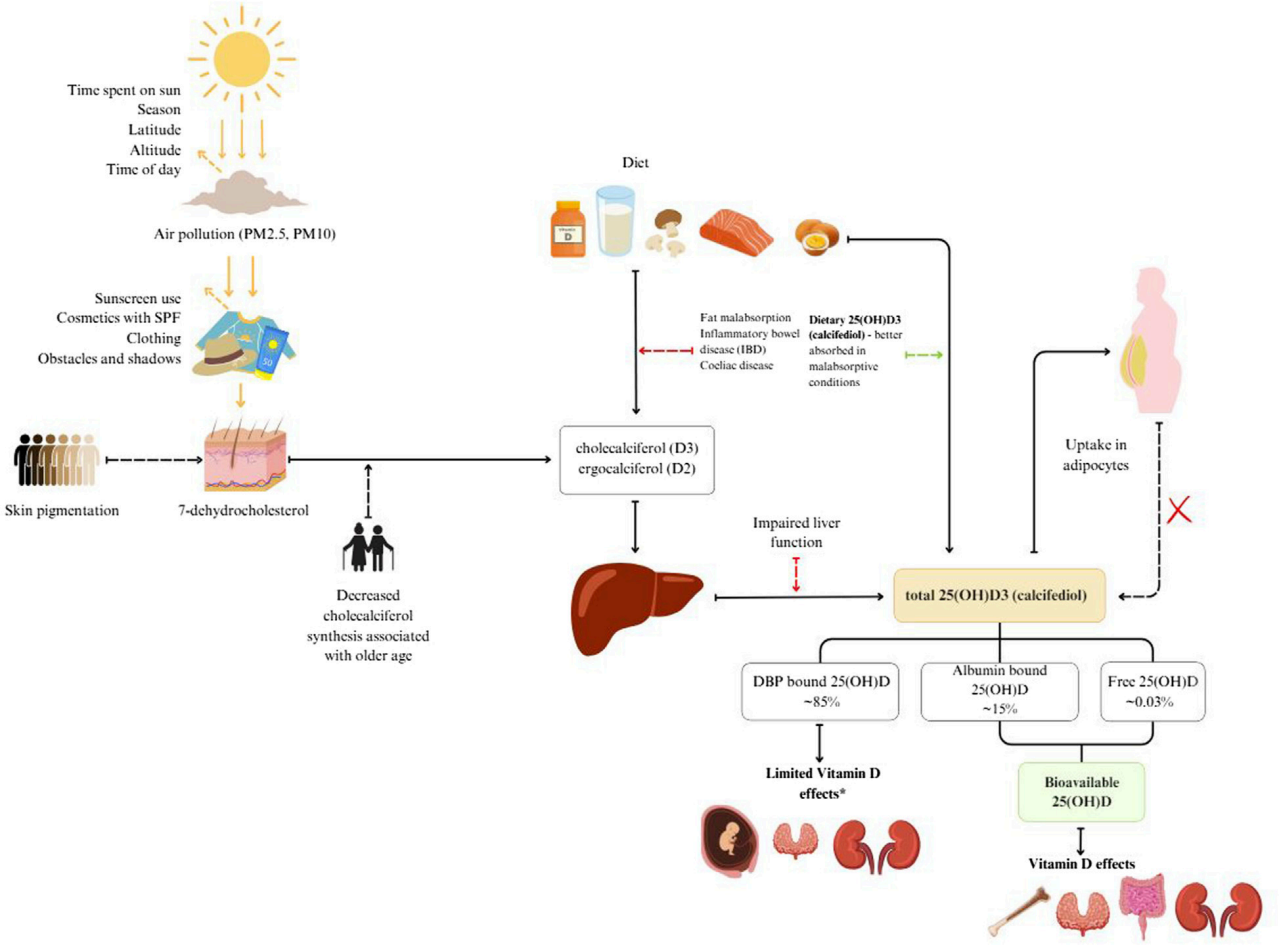
Vitamin D sources, metabolism and bioavailability.

Vitamin D sources, metabolism and bioavailability are schematically presented in Figure 1. Vitamin D is mainly obtained through diet and skin synthesis when exposed to UVB rays. Sun exposure is the primary source for most people, but without enough UVB exposure, dietary vitamin D becomes essential (Calvo et al., 2004; Spiro and Buttriss, 2014).

Vitamin D3 is produced from 7-dehydrocholesterol (7-DHC) in the skin through UVB radiation. It is converted to previtamin D3, which reaches peak concentration within hours (Holick, 2008a). This previtamin D3 is then transported to the liver via extracellular fluid and dermal capillaries, bound to vitamin D binding protein (DBP) (Holick et al., 2011).

Dietary sources of vitamin D include vitamin D2 (ergocalciferol), D3 (cholecalciferol), and 25-hydroxycholecalciferol [25(OH)D] (Taylor et al., 2014). These forms are transported to the liver in chylomicrons via lymph and blood plasma.

In the blood, two main fractions of vitamin D are measured. Total 25-hydroxyvitamin D (25(OH)D) is the primary circulating form and is often used as a marker for vitamin D status. It binds to DBP and is taken up by the kidneys through receptor-mediated endocytosis involving megalin and cubilin (Nykjaer et al., 2001). The active form, 1,25-dihydroxyvitamin D [1,25(OH)2D], is produced in the kidneys from 25(OH)D under the influence of parathyroid hormone (Lips, 2006; Holick, 2008b). This active form binds to vitamin D receptors, influencing calcium and phosphorus metabolism (Holick, 2008a; Bikle, 2014).

Vitamin D status typically refers to serum 25(OH)D concentration, which does not include vitamin D stored in fat or other tissues (Prentice et al., 2008; Bolland et al., 2018). This status is influenced by factors like body fat, muscle mass, and genetic variation (Cashman and Kiely, 2011; Ross et al., 2011).

Free 25(OH)D is the fraction that is not bound to DBP and can enter cells directly (Powe et al., 2013a). Bioavailable 25(OH)D includes free and albumin-bound 25(OH)D, making it more readily available for tissue uptake (Bikle and Schwartz, 2019).

DBP transports and stores vitamin D, increasing its stability and protecting it from degradation (Bikle, 2014; Speeckaert et al., 2006). Higher DBP concentrations increase vitamin D binding capacity, forming a larger reservoir of bound vitamin D (Smith and Goodman, 1971).

Measuring free and bioavailable vitamin D is complex and costly, making these measurements uncommon in clinical practice (Chun and Nielson, 2018). These can be calculated using total 25(OH)D, DBP, and albumin levels, although accuracy varies (Vičič et al., 2022).

Vitamin D status fluctuates with the seasons, especially at latitudes above 40°N where UVB exposure is insufficient during autumn and winter (Spiro and Buttriss, 2014; O’Neill et al., 2016).

### Seasonal variation

Sunlight-induced vitamin D synthesis in lighter-skinned populations begins at the end of March, with serum total 25(OH)D levels peaking after the summer months and then declining from October onwards. Therefore, a significant proportion of the European population relies on vitamin D from food and endogenous stores to maintain an adequate vitamin D status, especially during the winter season. A rather high prevalence of vitamin D deficiency has been reported in several countries (Spiro and Buttriss, 2014; O’Neill et al., 2016; Zittermann et al., 2016), and was also found in our previous studies (Hribar et al., 2020; Hribar et al., 2023). In addition to geographic location and season, there are other important factors that influence vitamin D status, such as age, sex, body mass index, constitutional skin color and various lifestyle factors (Webb et al., 2018; 1988; Hribar et al., 2020; Žmitek et al., 2020; Hribar et al., 2023).

The amount of solar radiation reaching an area is affected by the amount of time spent in the sun and factors that affect the angle of the sun: season, latitude, altitude and time of the day. Before sun rays reach the skin they can be blocked by particles in the air (PM_2.5_, PM_10_) and later by sunscreen use, cosmetics with SPF, clothing and obstacles such as buildings (Neville et al., 2021). When sun rays reach the skin they are partially blocked by melanin, which is dependent on skin pigmentation (Webb et al., 2018; Neville et al., 2021). In our previous study we showed that constitutive skin color, as well as tanning, hours spent in the sun and protective behaviors against the sun affect individual vitamin D levels (Hribar et al., 2023). Older age is associated with decreased cholecalciferol synthesis in the skin (MacLaughlin and Holick, 1985).

In some cases, taking vitamin D supplements may be recommended to maintain adequate levels of this important nutrient, especially during the winter months or in people who are at higher risk of deficiency (Lips, 2001; Kimball et al., 2007).

Studies have shown that levels of both bioavailable vitamin D and free vitamin D vary seasonally, with higher levels generally observed in the summer months and lower levels observed in the winter months (Zerwekh, 2008; Lehmann et al., 2013).

### Aims

The aim of the study was to determine and evaluate the relationship between the total 25(OH)D concentration, the amount of free and bioavailable vitamin D and the level of vitamin D binding protein (DBP) in Slovenian adults at the end of winter and at the end of summer.

## Materials and methods

### Study participants

Our study included 87 healthy volunteers aged 35.95 ± 12.55 years in the range (19–70 years), and the male-to-female ratio was 14:73. General information is presented in Table 1. The participants were part of the Nutri-D study: “Challenges in achieving adequate Vitamin D status in the adult population.” For this study a sub-sample of the Nutri-D study participants recruited in 2020 (the second year of the study) was used. The study was conducted with two observation periods, one in winter (January–February 2020) and one after the end of summer (September 2020). The invitation to participate in the study was posted on social media and the website of the Nutrition Institute (Slovenia).

**TABLE 1:** Population characteristics, vitamin D status of healthy Slovenian adults aged 19–70 years. The study was conducted in winter (January–February 2020) and after the end of summer (September 2020).

Variable	Category/Unit			
Age at study entry	years	35.95 ± 12.55		
Sex	♀ ♂	♀ 73 (83.9%) ♂14 (16.1%)		

Variable	Category/Unit	Winter n = 87	Summer n = 87	*p*-value
Total 25(OH)D	nmol/L	44.13 ± 17.82	74.97 ± 22.75	**<0.001**
	Range	10–84	28–166	
	<30	25.3%	2.3%	
	30–50	37.9%	4.6%	
	50–75	31.0%	54.0%	
	>75	5.7%	39.1%	
DBP	mg/L	239.86 ± 141.9	236.2 ± 164.39	0.768
	Range	38.7–853.1	31.6–967.2	
Albumin	g/L	47.97 ± 3.91	49.37 ± 4.15	**0.003**
	Range	38–60	41–64	
Bioavailable 25(OH)D (Vermulen)	nmol/L	7.45 ± 5.66	13.11 ± 8.27	**<0.001**
	Range	0.9–35.1	2.14–60.95	
Percentage of bioavailable 25(OH)D	(%)	16.9 ± 12.8	17.49 ± 11.03	
Free 25(OH)D	pmol/L	17.3 ± 12.9	29.7 ± 19.1	**<0.0001**

DBP, vitamin D binding protein. All values are presented as Mean ± SD or %. Values are presented as mean ± SD, *p* < 0.05 was considered statistically significant (*p* values of significant variables are in bold print).

Caucasian race (Fitzpatrick skin types I–IV), age over 18 years, desire to abstain from artificial UVB sources, and willingness to adhere to all study protocols were the inclusion criteria. Pregnancy or breastfeeding, severe sun avoidance (e.g., sun allergy), use of sunbeds, use of supplements or medications containing vitamin D, fish oil, or omega-3 fatty acids in the three months prior to study enrolment, regular (daily) consumption of foods enriched with vitamin D (e.g., fortified margarine or plant-based milk alternatives), adherence to a diet prescribed by a dietitian or medical staff, adherence to special diets (e.g., vegetarianism, low-carbohydrate, high-fat, and calorie restriction); current diseases of the kidneys, thyroid, digestive tract, osteoporosis and other bone diseases, skin diseases and other conditions that interfere with the absorption and synthesis of vitamin D, were the exclusion criteria for the study.

The study protocol was carried out in compliance with institutional and local regulations and was approved by the Ethics Committee of the Faculty of Applied Sciences (VIST), SI-1000 Ljubljana, Slovenia (Approval No. 2018/4-ET-SK). It was also registered at ClinicalTrials.gov (ID: NCT03818594). To take part, each subject had to complete an informed consent form.

### Methods of determination

In winter (January-February 2020) and after the end of summer (September 2020) a biochemical blood tube (4 mL) was collected. Albumin and total 25(OH)D were analyzed from fresh serum after blood collection, while the aliquot for DBP was stored at minus 80°C until analysis and all samples were analyzed simultaneously. Special care was taken to ensure that samples were taken from the same subject in the same batch on the same plate.

Measurements were performed at the Clinical Institute of Clinical Chemistry and Biochemistry (University Medical Centre, Ljubljana). Serum 25(OH)D, albumin and DBP, were measured in all participants using the following methods: vitamin 25(OH)D concentration was measured using a competitive luminescence immunoassay with a limit of quantification of 6 nmol/L (Architect analyzer, Abbott Diagnostics, Lake Forest, United States), Human Vitamin D Binding Protein was measured with ELISA (MyBioSource, Inc., San Diego, CA, United States); the limit of quantification was 31 mg/L. The concentration of albumin was measured with an automated Albumin Assay (ADVIA analyzer, Siemens, New York, United States). The assay is based on the reaction of albumin with bromcresol green followed by spectrophotometric detection; the limit of quantification was 10 g/L.

Specific formulas that use the amounts of total 25-hydroxyvitamin D [25(OH)D], vitamin D-binding protein (DBP), and albumin are used to calculate the bioavailable and free fractions of vitamin D. Predictive equations derived from equilibrium dialysis or ultrafiltration procedures may also be used to approximate these formulas.

### Calculation of free vitamin D

Free vitamin D can be calculated using the following formula:

$$\left[\text{Free }25\left(\text{OH}\right)D\right]=\left[\text{Total }25\left(\text{OH}\right)D\right]/\left\{1+\left(K_{\mathrm{DBP}}\times \left[\mathrm{DBP}\right]\right)+\left(K_{\mathrm{Alb}}\times \left[\mathrm{Alb}\right]\right)\right\}$$

Where:

• [Total [25(OH)D] is the total concentration of 25-hydroxyvitamin D.
• [DBP] is the concentration of vitamin D binding protein.
• [Alb] is the concentration of albumin.
• *K_DBP_* is the affinity constant of 25(OH)D for DBP (approximately 5.98 × 10^8^ M^−1^).
• *K* *_Alb_* is the affinity constant of 25(OH)D for albumin (approximately 6 × 10^5^ M^−1^).

### Calculation of bioavailable vitamin D

Bioavailable vitamin D includes both the free and albumin-bound fractions. It can be calculated as follows:

$$\left[\text{Bioavailable }25\left(\text{OH}\right)D\right]=\left[\text{Free }25\left(\text{OH}\right)D\right]+\left\{\left[\text{Total }25\left(\text{OH}\right)D\right]/\text{×}\;\left(1+K_{\mathrm{DBP}}\times \left[\mathrm{DBP}\right])+\left(K_{\mathrm{Alb}}\times \left[\mathrm{Alb}\right]\right)\times \left(K_{\mathrm{Alb}}\times \left[\mathrm{Alb}\right]\right)\right)\right\}$$

Simplified, the bioavailable vitamin D formula can be written as:

$$\left[\text{Bioavailable}25\left(\text{OH}\right)D\right]=\left[\text{Total}25\left(\text{OH}\right)D\right]/\left(1+\left(K_{\mathrm{DBP}}\times \left[\mathrm{DBP}\right]\right)+\left(K_{\mathrm{Alb}}\times \left[\mathrm{Alb}\right]\right)\right)\left(1+\left(K_{\mathrm{Alb}}\times \left[\mathrm{Alb}\right]\right)\right)$$

Free and bioavailable 25(OH)D were calculated using an online calculator (Vičič et al., 2022) based on a modified Vermeulen equation (Vermeulen et al., 1999; Powe et al., 2013a; Bikle et al., 2017).

### Statistical methods

The observed variables in the statistical analysis were total 25(OH)D, DBP, albumin, calculated bioavailable 25(OH)D and calculated free 25(OH)D. The explanatory variables were season (winter/summer), sex, and age.

The Endocrine Society cut-off values were used to assess total serum 25(OH)D levels target concentration for the optimal vitamin D effect: 75–125 nmol/L, insufficiency: 50–75 nmol/L and deficiency: <50 nmol/L (Holick et al., 2011; Holick et al., 2012; Vieth and Holick, 2018).

A paired sample *t*-test was performed to analyze differences between groups.

Values are expressed as mean ± SD or percentage (%) in the case of categorical variables. Statistical analysis was performed using SPSS, version 27 and MS Excel 2019.

A Pearson correlation coefficient was calculated to assess the linear relationship (Mukaka, 2012).

Odds ratios (OR) were calculated using binary logistic regression. For OR, 95% confidence intervals were calculated. The significance level was set at *p* < 0.05.

## Results and discussion

Our results show that vitamin D levels are lower in the winter months than in the summer months, that serum albumin concentrations are stable, and that DBP remains almost unchanged. Free vitamin D and bioavailable vitamin D levels are higher in the summer compared to the winter.

Total vitamin D levels are significantly lower in winter 44.13 ± 17.82 nmol/L compared to summer 74.97 ± 22.75 nmol/L (*p* < 0.001) (This is likely due to reduced sun exposure and lower cutaneous vitamin D synthesis during the winter, as supported by previous studies (Hribar et al., 2020; Hribar et al., 2023).

Although there is a small but significant increase in serum albumin concentration from winter 47.97 ± 3.91 g/L to summer 49.37 ± 4.15 g/L (*p* < 0.01), this change is not large enough to explain the year-round stability of vitamin D levels.

There was no significant difference in DBP levels from winter 239.86 ± 141.9 mg/L to summer 236.2 ± 164.39 mg/L (*p* = 0.77). This suggests that DBP is not a major factor in the seasonal variation of vitamin D levels.

There is a noticeable and significant increase in the free fraction of vitamin D from winter 17.3 ± 12.9 pmol/L to summer 29.7 ± 19.1 pmol/L (*p* < 0.0001), indicating higher availability of unbound vitamin D during the summer months.

The calculated bioavailable 25(OH)D is significantly lower in winter 7.45 ± 5.66 nmol/L compared to summer 13.11 ± 8.27 nmol/L (*p* < 0.001). This highlights the reduced availability of vitamin D for physiological use during the winter months.

The proportion of bioavailable 25(OH)D as a percentage of the total does not show a statistically significant difference between winter 16.9% ± 12.8% and summer 17.5% ± 11.0%. This suggests that while the absolute levels of bioavailable vitamin D vary seasonally, the relative proportion of bioavailable vitamin D to total vitamin D remains stable throughout the year.

These findings underscore the significant seasonal variation in vitamin D status, which is primarily driven by changes in sun exposure and subsequent synthesis of vitamin D. The stability of the proportion of bioavailable vitamin D relative to total vitamin D suggests that the body’s mechanisms for maintaining vitamin D homeostasis are robust, even though absolute levels fluctuate with the seasons.

**FIGURE 2:**
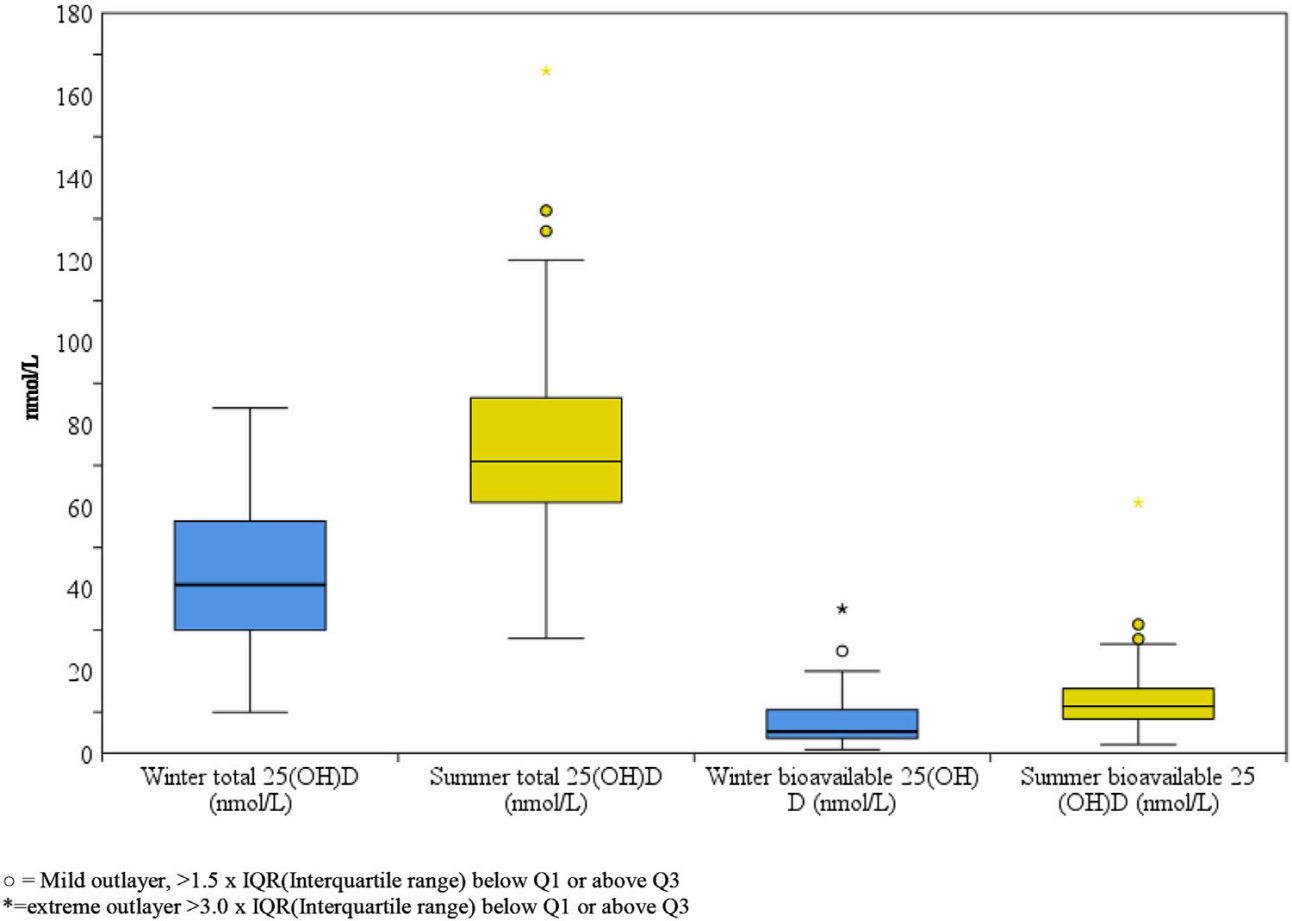
Box-plot comparison of winter and summer total and bioavailable 25(OH)D of healthy Slovenian adults aged 19–70 years. Study was carried out in winter (January-February 2020) and after the end of summer (September 2020).

A box plot of the winter and summer total 25(OH)D data and the estimated values of the bioavailable percentage are displayed in Figure 2.

### Correlation analysis

As part of our study, we also calculated the correlation between summer and winter total 25(OH)D and bioavailable vitamin results. The results are shown in Table 2.

**TABLE 2:** Pearson correlation coefficients (r) of blood biomarkers of healthy Slovenian adults aged 19–70 years. The study was conducted in winter (January–February 2020) and after the end of summer (September 2020).

		Bioavailable 25(OH)D		Total 25(OH)D	
		Winter	Summer	Winter	Summer
Total 25(OH)D	Winter	0.680*	0.370*	1	0.568*
	Summer	0.307*	0.343*	0.568*	1
Bioavailable 25(OH)D	Winter	1	0.509*	0.680*	0.307*
	Summer	0.509*	1	0.370*	0.343*

*Correlation is significant at the 0.01 level (2-tailed).

Wintertime Correlation of Total and Bioavailable 25(OH)D (*p* = 0.680): The robust association suggests that the total 25(OH)D levels are a reliable indicator of the bioavailable 25(OH)D levels during the winter. This suggests that in the absence of considerable sunlight the body’s regulatory systems may be at work to maintain a stable percentage of bioavailable vitamin D from the total amount available.

Winter total 25(OH)D and summer bioavailable 25(OH)D are correlated (*p* = 0.370): The fact that the association is smaller suggests that winter total 25(OH)D levels do not predict summer bioavailable 25(OH)D levels as well. Prediction based solely on winter levels is reduced because of seasonal variations and greater sun exposure in the summer, which probably result in additional factors influencing vitamin D bioavailability.

Summertime Correlation of Total and Bioavailable 25(OH)D (*p* = 0.343): Summertime correlations are similar to those observed in winter in that they are weak, meaning that although total 25(OH)D levels do affect bioavailable 25(OH)D levels, the effect is not as great. This decreased correlation may be caused by both increased sun exposure and perhaps greater diversity in the sun exposure habits of each subject.

Summer total 25(OH)D and winter bioavailable 25(OH)D are correlated (*p* = 0.307): Summer total 25(OH)D levels appear to be a poor predictor of winter bioavailable 25(OH)D levels, based on this weak association. This is to be expected given the stark variations in sun exposure, which cause the amounts of total and bioavailable vitamin D to change dramatically.

**FIGURE 3:**
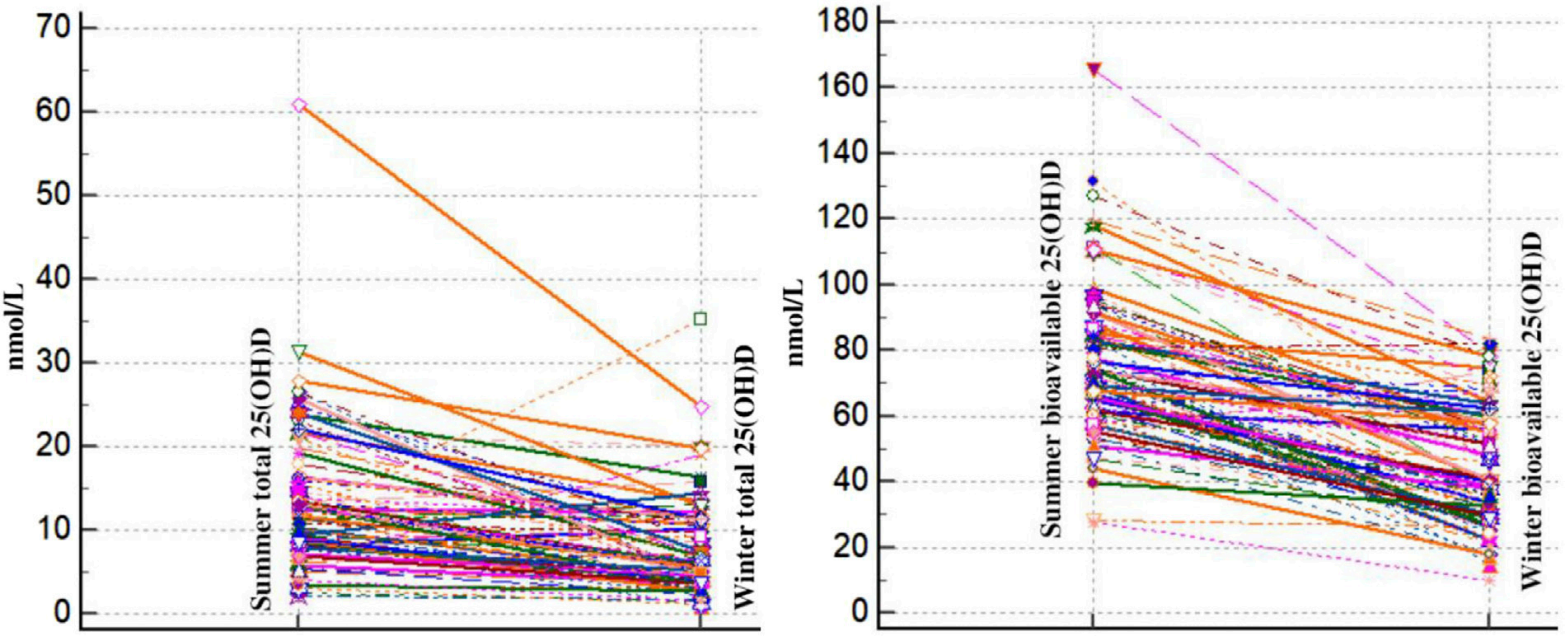
Spaghetti plot comparison of winter and summer total and bioavailable 25(OH)D of healthy Slovenian adults aged 19–70 years. Study was carried out in winter (January–February 2020) and after the end of summer (September 2020).

In addition, we looked at how the concentrations of total 25(OH)D and bioavailable vitamin D varied with the seasons. The results are shown in Figure 3.

From winter to summer, there is a noticeable and constant increase in total and bioavailable 25(OH)D levels in all individuals. This seasonal change makes sense given the well-documented consequences of increased sun exposure in the summer. There is observable individual diversity in the absolute levels of total and bioavailable 25(OH)D as well as the degree of growth, despite the general trend. This diversity can be explained by variations in the amount of time that each person spends in the sun, skin type, food consumption, and even hereditary variables that affect the metabolism of vitamin D. Across all the individuals, the proportionate increase in bioavailable 25(OH)D relative to total 25(OH)D appears to remain fairly constant. This implies that the mechanisms controlling the conversion and availability of bioavailable vitamin D are operating consistently in response to the elevated total vitamin D levels from summer sun exposure.

### Seasonal variation of total 25(OH)D levels

According to our research, the concentration of vitamin 25(OH)D is considerably lower in the winter than it is in the summer (Hribar et al., 2023). This significant seasonal variation is in line with earlier studies showing that seasonal variations in sunlight exposure have an impact on vitamin D levels. Several studies (Hine and Roberts, 1994; Kull et al., 2009; Hribar et al., 2023) have shown comparable trends and linked lower winter levels to decreased ultraviolet B (UVB) radiation. UVB light is necessary for the production of vitamin D in the skin.

### Serum albumin concentrations

Serum albumin concentrations varied significantly between the winter and summer (*p* = 0.003), but the magnitude of the variation was small. Higher summer levels of free and accessible vitamin D may be due in part to albumin, a key protein that binds to vitamin D. The small magnitude of variation, however, suggests that albumin alone has a limited effect on the seasonal variations in vitamin D levels.

### Vitamin D binding protein (DBP) levels

According to our research, summertime vitamin D binding protein levels are slightly lower than wintertime levels, but the difference is not statistically significant (*p* = 0.77). This is consistent with other research, including the findings of Powe et al. (2013b) that DBP levels do not significantly fluctuate with the seasons. The stability of DBP levels indicates that variations in total 25(OH)D levels are more likely to be the cause of changes in free and bioavailable vitamin D than fluctuations in DBP.

### Free fraction of vitamin D

There was a noticeable increase in the percentage of free vitamin D from winter to summer (*p* < 0.0001). There may be more unbound vitamin D available throughout the summer due to this significant seasonal increase in free vitamin D. This result is in line with recent studies showing that summertime increases in free vitamin D levels are caused by increased cutaneous production and decreased binding to albumin and DBP.

### Bioavailable 25(OH)D levels

With a *p*-value <0.001, bioavailable 25(OH)D was considerably lower in the winter than in the summer. This notable seasonal variation highlights the influence of a lack of UVB exposure on vitamin D bioavailability. The decrease in bioavailable vitamin D during the winter months may have effects on the immune system and bone health. Bioavailable vitamin D is essential for physiological processes and includes both free and albumin-bound fractions. These findings support research by several researchers (Bikle et al., 1986; Bouillon and Van Assche, 1995; O’Mahony et al., 2011; Charoenngam and Holick, 2020), highlighting the importance of bioavailable vitamin D in maintaining health.

### Proportion of bioavailable 25(OH)D as a percentage of total

Research such as that of Chun et al. (2010), which addresses the homeostatic control of vitamin D bioavailability, supports the relatively stable proportion of bioavailable 25(OH)D as a percentage of total vitamin D (± standard deviation), with 16.9% ± 12.8% in winter and 17.5% ± 11.0% in summer, which is not a statistically significant difference. This stability suggests that while absolute levels of bioavailable vitamin D vary seasonally, the body’s regulatory mechanisms maintain a consistent proportion of bioavailable to total vitamin D.

### Correlation between total 25-hydroxyvitamin D and bioavailable 25-hydroxyvitamin D levels in winter and summer

Because there are fewer daylight hours and less direct sunlight during the winter, there is less exposure to UVB irradiation of sufficient intensity to induce endogenous vitamin D synthesis. As a result, in order to maintain their vitamin D levels, humans rely primarily on food sources and stored vitamin D (from summer sun exposure). Both newly synthesized and stored vitamin D from food sources are included in total 25(OH)D. Conversely, vitamin D that is attached to albumin and the free fraction of vitamin D are known as bioavailable vitamin D, and these are readily absorbed by cells. Consequently, it is expected that a higher level of bioavailable 25(OH)D would correlate with a higher level of total 25(OH)D. This may be because, in the absence of substantial vitamin D deficiency, part of the stored vitamin D becomes accessible.

Summertime is when humans in our latitudes can produce extra vitamin D in their skin due to increased sun exposure and a higher intensity of UVB irradiation. When more freshly generated vitamin D from sunlight is available in the summer, the weak correlation seen in the winter may become a slightly positive correlation. The amount of stored vitamin D that contributes to total 25(OH)D levels decreases as more vitamin D is produced in the skin during the summer. The association with bioavailable vitamin D may be reduced as a result.

The higher correlation in winter indicates that the body is more dependent on its stored vitamin D at times when vitamin D availability is reduced. On the other hand, the reduced summer correlation may be a result of higher synthesis rates and distinct binding dynamics during periods of increased vitamin D availability.

A study of healthy adults in Switzerland, for example, found that both total and free vitamin D levels were significantly lower in winter than in summer, while there were no seasonal differences in bioavailable vitamin D levels (Merlo et al., 2015). In contrast, a study of older adults in the United Kingdom found that while total vitamin D levels were lower in winter, there was no significant difference in free or bioavailable vitamin D between summer and winter months (Cashman et al., 2012; Hilger et al., 2014), Vitamin D supplementation may increase total vitamin D levels, including the bioavailable fraction. However, the extent to which supplementation affects the bioavailable fraction may depend on several factors, including the form and dose of vitamin D, individual differences in vitamin D metabolism and absorption, and the presence of underlying medical conditions.

A study conducted on postmenopausal women with low vitamin D levels found that supplementation with vitamin D_3_ significantly increased both total and bioavailable vitamin D levels (Hassanein et al., 2023). Another study of overweight and obese adults with vitamin D deficiency showed that daily supplementation with high-dose vitamin D_3_ for 16 weeks increased bioavailable vitamin D levels (Entezari et al., 2018).

However, some studies have also reported that vitamin D supplementation does not increase bioavailable levels equally in all individuals. For example, one study of healthy adults found that 12 weeks of vitamin D_3_ supplementation did not significantly alter bioavailable vitamin D levels (Jorde and Grimnes, 2011; Chandler et al., 2015; Flueck et al., 2016).

Food fortification with vitamin D can be an effective strategy for increasing vitamin D intake in populations with low sun exposure or inadequate dietary intake.

Possibilities for food fortification with vitamin D are numerous Examples include but are not limited to: milk, fat spreads (Jääskeläinen et al., 2017), milk alternatives such as soy beverages (Vatanparast et al., 2020), orange juice (Tangpricha et al., 2003), flour and cereals (Allen et al., 2015), UV irradiated mushrooms (Cardwell et al., 2018), UV-irradiated yeast for the preparation of bread (EFSA Panel on Dietetic Products Nutrition and Allergies NDA, 2014), and biofortification of chicken eggs (Browning and Cowieson, 2014; Vičič et al., 2023). The media for fortification should be consumed regularly by the majority of the population and should minimally increase production costs and prices for consumers (Pilz et al., 2018).

Reliable total 25(OH)D, DBP, and albumin levels are necessary for calculation accuracy. Laboratory procedures and assays can differ, which can impact the outcomes.

Genetic variants and individual variations in DBP isoforms can affect binding affinities and concentrations, which in turn can affect the estimated levels of free and bioavailable 25(OH)D (Fernando et al., 2020).

Although helpful, the formulas for determining the free and available fractions of vitamin D have some drawbacks. These include assumptions regarding individual biological differences, measurement variability in proteins, and binding constants.

Immunoassays made specifically to measure free 25-hydroxyvitamin D [25(OH)D], equilibrium dialysis, and ultrafiltration are direct methods for determining levels of free and bioavailable vitamin D. These techniques directly measure the unbound and albumin-bound fractions of vitamin D, with the goal of giving a more realistic picture of the physiologically active forms of the vitamin but their complexity and resource needs prevent frequent use, and are useful in research contexts and for detailed clinical assessments.

### Study limitations and future research

The use of bioavailable 25(OH)D or free 25(OH)D may be useful in physiological and pathological conditions that affect DBP, such as pregnancy, genetic polymorphisms, liver disease, and kidney disease (Tsuprykov et al., 2018a), when comparing vitamin D status in premenopausal and postmenopausal women (Vičič et al., 2022) and in ethnically diverse populations (Aloia et al., 2015; Tsuprykov et al., 2018a; Abidin and Mitra, 2020). There is also a discrepancy between calculated free 25(OH)D and directly measured free 25(OH)D. This is more pronounced in African Americans and has been attributed to differences in DBP binding capacity between ethnic groups (Schwartz et al., 2014). Currently there are no generally accepted reference values for calculated or directly measured free and bioavailable 25(OH)D. Zeng et al. (2021) established reference values for free 25(OH)D through linear regression models but emphasized the need for clinical studies to validate their recommendations. Tsuprykov et al. (2018b) established different reference values in pregnant women for both calculated and measured free 25(OH)D. They concluded that both methods may be appropriate.

We did not evaluate bone health or markers of other significant diseases to determine whether bioavailable/free 25(OH)D is a better predictor of low bone mass density and other conditions. We are currently focusing on this assessment in patients with liver disease.

## Conclusion

Calculating vitamin D fractions using formulas is a cost-effective and convenient alternative when direct measurement of individual fractions is not available or feasible. However, it is important to consider the assumptions, accuracy and variability associated with the formulas used and recognize that direct measurement methods may provide more accurate and reliable results.

Our study provides important insights into seasonal variations in vitamin D status and bioavailability. We found that vitamin 25(OH)D concentrations are significantly lower in winter compared to summer, with a slight, non-significant difference in vitamin D binding protein from summer to winter, and significantly higher albumin concentrations in summer. Both bioavailable and free vitamin D levels are significantly lower in winter.

The primary cause of the winter decrease in total 25(OH)D levels is reduced sunlight exposure, which limits vitamin D synthesis. This also leads to reduced bioavailable and free vitamin D, indicating that not only is less vitamin D produced, but the amount available for use by the body is also reduced. The stable DBP levels suggest that DBP is not the main factor in the seasonal variation of free and bioavailable vitamin D. The seasonal increase in albumin concentration during summer may contribute to a greater amount of bioavailable vitamin D. Albumin’s weaker binding affinity for vitamin D compared to DBP allows for easier dissociation of vitamin D, shifting the balance toward more free and albumin-bound vitamin D available for cellular uptake.

There is a moderately positive correlation between total and bioavailable 25(OH)D in winter and a lower positive correlation in summer. This stronger winter correlation suggests that during low vitamin D availability, the body relies more on existing stores, while the weaker summer correlation indicates that with higher vitamin D synthesis, bioavailability dynamics are less dependent on total vitamin D levels.

## Data Availability

The raw data supporting the conclusions of this article will be made available by the authors, without undue reservation.

## Abbreviations

BMI, body mass index; UV, ultraviolet; D3, cholecalciferol; D2, ergocalciferol; Total 25(OH)D, 25-hydroxycholecalciferol, calcifediol, calcidiol, 25-hydroxyvitamin D3; 1,25-(OH)D, 1,25-dihydroxyvitamin D; DBP, vitamin D binding protein; OR, Odds ratio.
